# Simple and Safe Packing Method for High-Grade Liver Injuries

**DOI:** 10.5812/atr.5301

**Published:** 2012-06-01

**Authors:** Mehrdad Hosseinpour, Mohammad Reza Asgarzadeh, Mahdi Mohammadzadeh, Farzad Parvizian

**Affiliations:** 1 Trauma Research Center, Kashan University of Medical Sciences, Kashan, IR Iran; 2 Department of Surgery, Faculty of Medicine, Isfahan University of Medical Sciences, Isfahan, IR Iran

**Keywords:** Liver, Wounds and Injuries, Bleeding

## Abstract

**Background:**

Injury to the liver is a commonly encountered problem in trauma cases and is a frequent cause of morbidity and mortality. Because gauze packing is easy to use and has the potential for rapid hemorrhage control, it is the most commonly used method for patients with severe liver injuries, particularly those with coagulopathy.

**Objectives:**

In this study, OpSite sheets were used to make three-layer packs for decreasing the complication associated with removing gauze packing.

**Patients and Methods:**

Twenty male patients with grade IV or V liver injuries that required laparotomy were enrolled in the study. Ten patients were treated using conventional packing, while the other 10 were treated using the three-layer pack. In the case group, the liver was mobilized as much as possible. The three-layer pack was then placed at the site of liver damage and extended onto the liver surface, and the other pads were placed on top of this pad. After 72 h, reoperation was performed, the packs were removed, and the packs causing injury were recorded. Additionally, if rebleeding due to the adhesive bands of the pack was observed, the blood was suctioned and bleeding volume was measured. Data were analyzed using the Mann–Whitney test.

**Results:**

Patients in the case and control groups were similar in age and admission vital signs. During the second operation, the bleeding volumes measured in the case and control groups were 66 ± 27.01 mL and 152 ± 85.4 mL, respectively. There was some pad-induced damage after the removal of the pad in the control group.

**Conclusions:**

Our study has provided a simple and safe packing method for high-grade liver injuries.

## 1. Background

The liver is the largest solid abdominal organ, and its relatively fixed position makes it prone to injury. It is the second most commonly injured organ in abdominal trauma, and its damage is among the most common causes of death after abdominal trauma (1). Mortality rates of patients with grade IV and V injuries have been estimated to range between 35% and 80% (2, 3). Non-operative treatment of the isolated hepatic injury in the stable patient is now considered a standard practice (4-5). However, various surgical techniques such as packing, anatomic liver resection, and total hepatectomy with liver transplantation can be used to manage unstable patients with liver injury (2, 6-10). Because gauze packing is easy to use and has the potential for rapid hemorrhage control, it seems to be the most commonly used method for treating patients with severe liver injuries, particularly those with coagulopathy (11). This method, however, also has several disadvantages, including the need to perform a reoperation to remove the packing, as well as rebleeding.

## 2. Objectives

In this study, OpSite sheets were used to make three-layer packs to decrease the complications that are encountered when removing gauze packing.

## 3. Patients and Methods

This clinical trial investigated the use of a three-layer pack to decrease the amount of bleeding that occurs during pack removal in adult male trauma patients. The study was approved by the Isfahan University of Medical Sciences Ethics Committee and conducted from January 2007 to January 2008.

**Figure 1.**
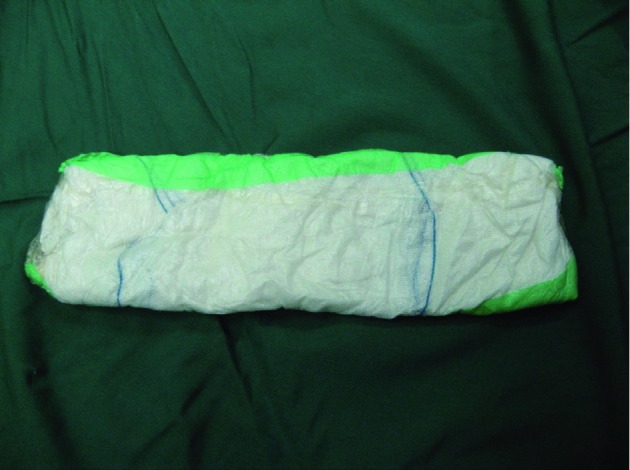
Three-layer pack

Twenty male patients with grade IV or V liver injuries that required laparotomy were enrolled in the study. The main indication of surgery for the patients was the instability of vital signs after proper infusion of intravenous fluids and blood transfusion. For comparison, we divided the study group into 2 subgroups (10 patients as cases and 10 as controls). Patients were transported to the operating room, where the midline abdominal incision was explored. In all cases, significant liver disruption was documented. Patients who could be managed by other methods of liver repair were excluded; patients with the need for liver packing for bleeding control were enrolled in the study. In the control group, the liver was packed with the conventional method described by Feliciano *et al. *(4). In the case group, the liver was mobilized as much as possible. The three-layer pack was then placed on the site of liver damage and extended onto the liver surface. The three-layer pack is made of 1 sterile, long pad sandwiched between 2 layers of OpSite sheet (Smith & Nephew Healthcare Company) arranged bilaterally *(Figure 1)*. After placing the three-layer pack on the liver, 3–4 long pads were placed on top of the three-layer pack to maintain pressure on the liver surface. In this study, we did not use procoagulant tissue adhesive or fibrin glue. After closure of the abdomen, the patients were observed in the intensive care unit for a period of 72 h. After 27 h, the reoperation was performed; the packs were removed, and the packs that induced injury were recorded. Additionally, if rebleeding due to the adhesive bands of the packs was observed, the blood was suctioned and the volume of blood in the suction bottle as well as the blood on the swabs was measured. Data abstractors recorded demographic data, initial vital signs (systolic and diastolic blood pressure, pulse rate, etc.), and the mechanism of trauma for all patients. Upon completion of the trauma workup, an injury severity score (ISS) was calculated. We also recorded the volume of bleeding during the second operation. The data are reported as means ± standard deviations. The Mann–Whitney test was used to analyze continuous variables for determining the differences between the groups. Calculations were performed using SPSS 11.5 (SPSS Inc., Chicago). *P *values < 0.05 were considered significant. 

## 4. Results

During the study period, 29 male patients met the enrollment criteria (i.e., suspected major liver injury and the requirement for an operative procedure). Nine patients who required liver parenchymal repair were excluded. The final analysis was performed on 20 patients (10 cases and 10 controls). The mean age was 36 ± 16 years (range, 20–57 years). Seven patients (35%) had major injuries (ISS > 15). The mechanisms of injury, which included blunt trauma in all the patients, were as follows: motor vehicle crash (n = 4, 20%), pedestrian struck (n = 4, 20%), motorcycle crash (n = 4, 20%), and fall (n = 8, 40%). A comparison of the baseline variables among the study groups is presented in *Table 1*. Patients in the case and control groups were similar in age, admission vital signs, and ISS. During the second operation, bleeding volumes were 66 ± 27.01 mL (range, 30–100 mL) and 152 ± 85.4 mL (range, 90–300 mL) in the case and control groups, respectively (*P *= 0.016). There was some pad-induced damage (laceration) after the removal of the pad in the control group. In one patient in the control group, we had to repack the liver during the second operation. There were no derangements in liver function at the time of pack removal in either group. Patients were followed up for 3 months. Neither septic complications nor bile leaks were observed in the patients, and there was no mortality. 

**Table 1.** Characteristics of the Patients in the Case and Control Groups

	Cases, Mean ± SD	Controls, Mean ± SD
Age, y	37 ± 15	38 ± 14
Systolic blood pressure, mmHg	90.1 ± 20.2	80.8 ± 30
Diastolic blood pressure, mmHg	50.2 ± 20.4	50.1 ± 10.7
Heart rate, bpm	115 ± 8	117 ± 6.2
Blood loss in the first operation, L	1.3 ± 0.6	1.1 ± 0.82
Injury severity score	12.09 ± 10.15	14.17 ± 7.47
Total amount of blood, mL	1012 ± 430.8	1030 ± 623.2

## 5. Discussion

Liver injury has been cited as the most common cause of injury-associated death after abdominal trauma (3). Most solid organ injuries can be successfully controlled by conservative management and transfusion of blood-derived products. However, in the most severe cases, liver trauma presents a difficult clinical challenge that manifests as continued blood loss requiring subsequent resuscitation resulting in a clinical picture of coagulopathy, severe acidosis, and hypothermia (2, 11). Therefore, operation and bleeding control are still the procedures of choice for high-grade liver injuries. Liver packing was initially described by Feliciano *et al.* (4) for the control of bleeding, and to date, it is the standard approach for the control of damage in liver injuries in unstable patients. Although this technique has many advantages (12, 13), there are some difficulties encountered when the surgeon removes the packing. Adhesive bands between the liver damage area and the pad develop during the interoperation period, and the removal of the pad in the second operation can cause some disruption of the liver tissue and rebleeding. In some cases, rebleeding is so severe that it requires parenchymal repair with sutures (5, 13, 14). In this study, we used a new method for liver packing to decrease liver injury and rebleeding during the second operation. The OpSite sheet is made of a thin, polyurethane membrane that is coated with a layer of acrylic adhesive. The dressing, which is permeable to both water vapor and oxygen, is impermeable to microorganisms. Once in position, the OpSite sheet provides an effective barrier to external contamination, whilst producing a moist environment at the surface of the wound by reducing water vapor loss from the exposed tissue. The main use of this sheet is for wound dressing. To our knowledge, this is the first study that uses OpSite sheets for liver packing in trauma patients. Our findings have shown that there is a significant difference in the bleeding volume between the study groups. Our results are consistent with the findings of Sitzmann* et al*. (15), who used a non-stick bowel bag to wrap the liver surfaces and observed no liver rebleeding after the removal of the bag. Although their results showed the utility of the bowel bag, there were some limitations to their study. First, they completely mobilized the right lobe of the liver for complete wrapping, but this was not always possible. Moreover, this maneuver sometimes increases the injury to the liver. Second, they used a procoagulant tissue adhesive and fibrin glue on the raw liver surfaces, but these agents are not accessible in every trauma center. In contrast, our technique is simple and can be used in every operation room. The three-layer pack creates a smooth surface and a more manageable pressure than other pads. This could be an important advantage as shown by Koniais* et al*. (16), who have demonstrated that the regeneration ability of liver was decreased because of excessive packing pressure. In summary, our study has provided a simple and safe packing method for high-grade liver injuries.
